# Natural, Designed and Engineered Metalloenzymes: Structure, Catalytic Mechanisms and Applications

**DOI:** 10.3390/ijms241814255

**Published:** 2023-09-19

**Authors:** Linda Leone, Flavia Nastri, Angela Lombardi

**Affiliations:** Department of Chemical Sciences, University of Naples Federico II, 80126 Naples, Italy; linda.leone@unina.it (L.L.); flavia.nastri@unina.it (F.N.)

Bioinorganic chemists have become engaged in the challenge of elucidating the molecular mechanisms that govern how protein scaffolds modulate the properties of metal cofactors. One of the most powerful tools for this purpose consists of the construction of model systems replicating the structural and functional features of their natural counterparts. Metalloenzymes also represent a significant source of inspiration: Researchers have attempted for decades to construct efficient and selective catalysts by engineering metal binding sites into natural or de-novo-designed protein scaffolds. From this perspective, the construction of artificial metalloenzymes goes beyond the goal of elucidating the catalytic mechanism of natural biocatalysts and aims to manipulate or improve their catalytic properties into tailor-made systems.

This Special Issue of the International Journal of Molecular Sciences has collated some recent contributions involving the development of artificial metalloenzymes for challenging biocatalytic applications. Several strategies have been successfully applied to this purpose, and some prominent examples are reported herein.

The modification of natural metalloproteins is a widely applied approach for developing artificial biocatalysts. This can involve either the rational mutation of specific residues close to the metal center, or its replacement with an artificial metal cofactor. In this context, myoglobin (Mb) has been the preferred protein scaffold by a number of research groups, demonstrating itself to be highly tolerant to several modifications. Lin and coworkers designed a four-residue mutant of Mb to introduce the key structural features found in peroxidase-active sites, turning the O_2_-carrier protein into an efficient artificial peroxidase. The engineered enzyme can efficiently catalyze the decolorization of a variety of organic dyes and the bioconversion of Kraft lignin [1]. Hayashi and coworkers reconstituted apo-Mb with cobalt corrole (CoCor), affording an artificial catalyst able to activate hydrogen peroxide and promote the oxidation of phenolic substrates [2]. Detailed kinetic studies highlighted that the CoCor-substituted protein acts through a different mechanism compared to natural heme peroxidases, providing intriguing details for future designs. Another example of redesign altering protein functionality has been reported by Tan and coworkers, which converted the dinuclear nickel-dependent truncated acetyl-coenzyme A synthase into a superoxide dismutase (SOD) [3]. Altering the composition of the first coordination sphere at the distal nickel (Ni_d_) binding site provided the protein with SOD activity, but the modulation of second-sphere interactions was revealed to be crucial in enhancing the catalytic performances.

Further, de novo protein design offers the opportunity to completely shape the structural and metal binding properties of a peptide scaffold, which is constructed totally from scratch. Significant advances have been achieved in this field thanks to increased sophisticated computational tools now available, allowing for a precise prediction of protein structures. Following this path, Chackarborty and coworkers targeted the proximal nickel site (Ni_p_) of acetyl-coenzyme A synthase and engineered a Ni(Cys)_3_ binding site into a de-novo-designed three-stranded coiled coil [4]. α-Helical bundles are privileged scaffolds for engineering metal binding sites since they can tolerate multiple mutations without disrupting the global fold and can be designed with the highest degree of confidence. Mahy, Ricoux, and coworkers also exploited an artificial α-helical protein scaffold, named α-Rep, to construct a cobalt–porphyrin-containing enzyme for the CO_2_ reduction from neutral water [5]. This study highlights the importance of coupling experimental and theoretical approaches for elucidating the interaction between the cofactor and the host protein, also providing mechanistic insights. This aspect has also been emphasized by the study of Davari, Yildiz, and coworkers, who modeled the binding kinetics of active site inhibitors to the A disintegrin and metalloproteinase complex (ADAM17), involved in the acute inflammatory response [6]. Two α-helical peptides embracing a covalently bound Fe-porphyrin represent the minimal peptide scaffold of the mini-enzyme FeMC6*a. Nastri and coworkers have showcased the great catalytic potential of this miniaturized heme protein by showing its ability to oxidize several halogenated phenols with substrate-dependent chemoselectivity [7].

It must be underlined that several of the artificial metalloenzymes described in this Special Issue have been studied for environmental applications, including biomass valorization, CO_2_ reduction, and pollutant degradation. Moreover, two excellent review articles have been reported on related topics. Arrigoni, Bertini, and coworkers have presented an overview of theoretical investigations for elucidating the mechanism of metalloenzymes involved in the degradation and valorization of natural biopolymers and synthetic plastics [8]. Further, Leone, Lombardi, and coworkers have described the most recent advances in the development of homogeneous hydrogen evolution catalysts envisioned to mimic hydrogenases [9].

We hope that this Special Issue, outlining recent developments in the field of metalloenzymes design and engineering, highlights the rapid progress and long-term potential of these systems.
